# Systematic analysis of an evolved *Thermobifida fusca* muC producing malic acid on organic and inorganic nitrogen sources

**DOI:** 10.1038/srep30025

**Published:** 2016-07-18

**Authors:** Yu Deng, Jia Lin, Yin Mao, Xiaojuan Zhang

**Affiliations:** 1 National Engineering Laboratory for Cereal Fermentation Technology (NELCF), Jiangnan University, 1800 Lihu Road, Wuxi, Jiangsu 214122, China; 2 The Key Laboratory of Industrial Biotechnology, Ministry of Education, Jiangnan University, 1800 Lihu Road, Wuxi, Jiangsu 214122, China; 3 College of Life Science, North China University of Science and Technology, Tangshan 063000, China; 4 School of pharmaceutical science, Jiangnan University, 1800 Lihu Road, Wuxi, Jiangsu 214122, China

## Abstract

*Thermobifida fusca* is a thermophilic actinobacterium. *T. fusca* muC obtained by adaptive evolution preferred yeast extract to ammonium sulfate for accumulating malic acid and ammonium sulfate for cell growth. We did transcriptome analysis of *T. fusca* muC on Avicel and cellobiose with addition of ammonium sulfate or yeast extract, respectively by RNAseq. The transcriptional results indicate that ammonium sulfate induced the transcriptions of the genes related to carbohydrate metabolisms significantly more than yeast extract. Importantly, *Tfu_2487*, encoding histidine-containing protein (HPr), didn’t transcribe on yeast extract at all, while it transcribed highly on ammonium sulfate. In order to understand the impact of HPr on malate production and cell growth of the muC strain, we deleted *Tfu_2487* to get a mutant strain: muCΔ2487, which had 1.33 m**o**le/mole-glucose equivalent malate yield, much higher than that on yeast extract. We then developed an *E. coli*-T. fusca shuttle plasmid for over-expressing HPr in muCΔ2487, a strain without HPr background, forming the muCΔ2487S strain. The muCΔ2487S strain had a much lower malate yield but faster cell growth than the muC strain. The results of both mutant strains confirmed that HPr was the key regulatory protein for *T. fusca*’s metabolisms on nitrogen sources.

*Thermobifida fusca* is a thermophilic actinobacterium, which is an efficient degrader of plant cell walls^1^. *T. fusca* has been heavily studied for more than 30 years because it can produce a variety of cellulases, hemicellulases and other enzymes. These enzymes especially cellulases are thermostable and their activities retain high from pH 4 to 10^2^,^3^,^4^,^5^,^6^,^7^,^8^,^9^. Previously, a mutant strain, *T. fusca* muC obtained from adaptive evolution by cultivating *T. fusca* WT on non-lethal medium, was found to accumulate malic acid from different sugars^10^. Malic acid is an important value-added product of the C4 diacids family, reported by the U.S. Department of Energy as the chemical of great commercial interest produced from renewable substrates^11^. The malic acid synthesis pathway in *T. fusca* was identified by Deng *et al*.: phosphoenolpyruvate from glycolysis pathway was converted to oxaloacetate, which was then reduced to malate^12^.

Previously, the nitrogen sources were proved to affect the cell growth and malic acid production significantly^12^. In this study, we did transcriptome analysis of *T. fusca* muC on Avicel and cellobiose with addition of ammonium sulfate and yeast extract, respectively. The histidine phosphocarrier protein (Hpr or Tfu_2487) didn’t express at all in the muC strain on ammonium sulfate, with faster cell growth and less malic acid yield compared to the one on yeast extract. In order to understand the impact of HPr on the muC strain’s metabolisms, the *hpr* gene was deleted in *T. fusca* muC, forming muCΔ2487. *Tfu_2487* was then over-expressed in muCΔ2487, forming muCΔ2487S, which was grown on ammonium sulfate and yeast extract.

## Results

### Effect of nitrogen sources

**Figure 1:**
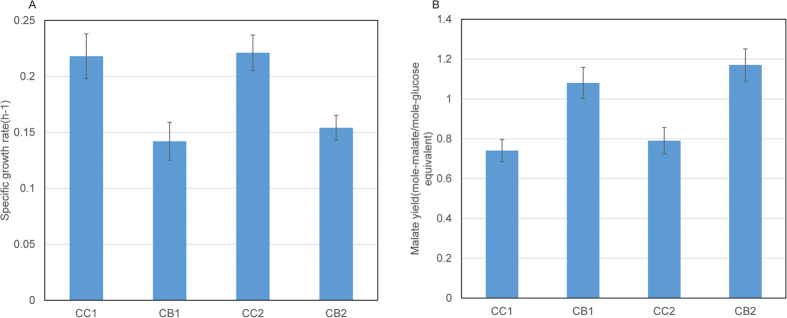
The fermentation results of *T. fusca* muC on different conditions. (**A**) The specific growth rate; (**B**) Malate yield. CB1: 2 g/L yeast extract, 5 g/L cellobiose; CB2: 2 g/L yeast extract, 5 g/L Avicel; CC1: 2 g/L ammonium sulfate, 5 g/L cellobiose; CC2: 2 g/L ammonium sulfate, 5 g/L Avicel.

The muC strain was grown on four conditions with different carbon and nitrogen sources: 1) CB1: 2 g/L yeast extract, 5 g/L cellobiose; 2) CB2: 2 g/L yeast extract, 5 g/L Avicel; 3) CC1: 2 g/L ammonium sulfate, 5 g/L cellobiose; 4) CC2: 2 g/L ammonium sulfate, 5 g/L Avicel. The fermentation results are shown in Fig. 1. The specific growth rates on CC1 and CC2 were 0.218 h^−1^ and 0.221 h^−1^, which were significantly higher than those on CB1 (0.142 h^−1^) and CB2(0.154 h^−1^). However, the muC strain on CB1 and CB2 had much higher malic acid yields than those on CC1 and CC2. It seems that yeast extract is much more suitable for producing malic acid, while *T. fusca* muC favors ammonium sulfate to accumulate biomass.

### Global gene expression and clusters of orthologous groups (COG) analysis

In order to investigate transcriptional changes on different conditions, the transcriptomes of the muC strain on CB1, CB2, CC1 and CC2 were analyzed by RNAseq. The RNAseq results were stored in the GenBank (SRA #: SRP067532). The results of transcriptomes are shown in Supplementary Table S2.

**Figure 2:**
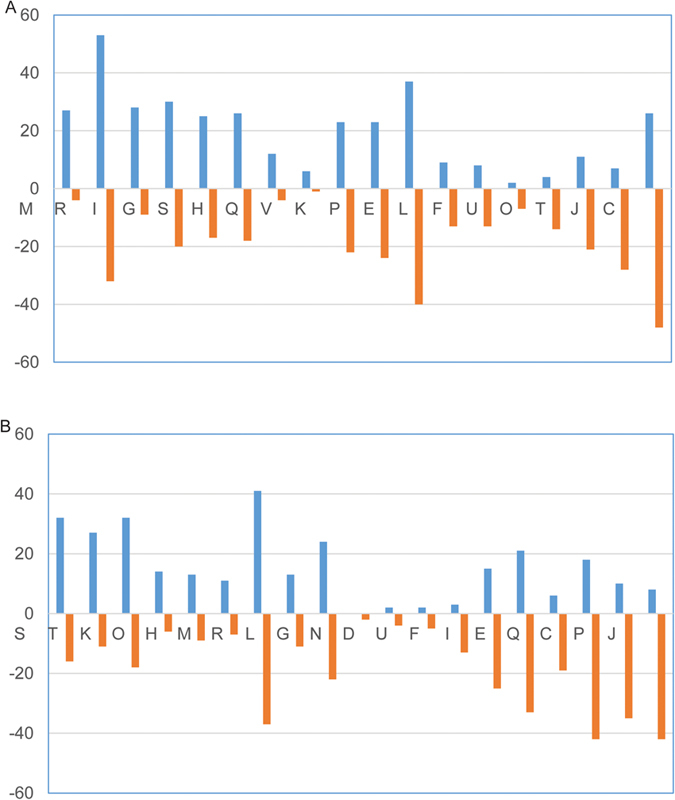
The clusters of orthologous groups (COG) distributions for the genes up-regulated and down-regulated in the transcriptomes. (**A**) CC1 vs. CB1; (**B**) CC2 vs. CB2.

The COG distributions for the genes up-regulated and down-regulated in the transcriptomes of them are shown in Fig. 2. Compared to CB1, *T. fusca* muC on CC1 had 357 genes up-regulated and 335 genes down-regulated; the top three categories of up-and down-regulated genes are: R: general function prediction only (equivalent to unclassified), E: Amino acid transport and metabolism and C: Energy production and conversion. Compared to CB2, *T. fusca* muC on CC2 had 292 genes up-regulated and 357 genes down-regulated. The top three categories of up-and down-regulated genes are: R: General function prediction only; C: Energy production and conversion; C: Amino acid transport and metabolism.

The observed cell growth and metabolite changes were phenotypes that largely depended on changes in carbohydrate transport and metabolism. The genes responsible for producing malic acid by muC were also mainly included in the above category. When considering genes associated with carbohydrate transport and metabolism, RNA transcripts in CC1 (as compared to the CB1 strain): 184 genes up-regulated and 27 genes down-regulated; the ones in CC2 (as compared to the CB2): 172 genes up-regulated and 10 genes down-regulated.

Because *T. fusca* muC on CC1 and CC2 used ammonium sulfate as the sole nitrogen source and *T. fusca* muC on CB1 and CB2 used yeast extract as the sole nitrogen source, the above transcriptional results indicate that ammonium sulfate induced the transcriptions of the genes related to carbohydrate metabolisms significantly more highly than yeast extract. Based on the cell growth in Fig. 1, it is indicated that the higher transcriptional levels of the genes related to carbohydrate metabolism were in agreement with the faster cell growth of the muC strain on CC1 and CC2 (ammonium sulfate as the nitrogen source).

### Genes related to cellulases

The widely accepted mechanism for enzymatic hydrolysis of cellulose involves the synergistic activity of endocellulases, exocellulases and processive cellulases^13^. There were 10 cellulase genes and 3 hemicellulase genes found to be highly expressed in the *muC* strain under all conditions (Table 1).

In Table 1, 7 out of 10 cellulase genes and 2 out of 3 hemicellulase genes of the muC strain on CC1 transcribed more than those on CB1(LFC ≥ 0). All of the cellulase and hemicellulase genes except *Tfu_1607* of the muC strain, transcribed significantly more on CC2 than those on CB2 (LFC ≥ 1).

**Figure 3:**
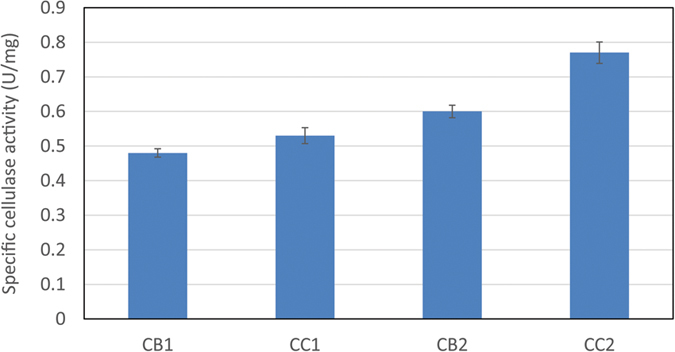
Cellulase activities on different conditions. CB1: 2 g/L yeast extract, 5 g/L cellobiose; CB2: 2 g/L yeast extract, 5 g/L Avicel; CC1: 2 g/L ammonium sulfate, 5 g/L cellobiose; CC2: 2 g/L ammonium sulfate, 5 g/L Avicel.

Although most of the cellulase gene’s expressions were higher on ammonium sulfate than on yeast extract, in order to estimate the synergistic effect of the cellulases corresponding to gene expressions, the specific cellulase activities were tested. In Fig. 3, the muC strain on CC1 had much higher specific cellulase activity than the one on CB1. And the specific cellulase activity of muC on CC2 was much higher than that on CB2. Thus, the cellulase gene expressions were in agreement with the specific cellulase activity well.

### Malic acid production

The synthesis of malic acid involved phosphoenolpyruvate carboxykinase (Tfu_0083) and malate dehydrogenase (Tfu_0092). The degradation of malic acid included ‘malic’ enzyme (Tfu_0562), malate dehydrogenase (oxaloacetate decarboxylating) (Tfu_2390) and fumarate hydratase (Tfu_0459)^12^. All the genes related to malic acid metabolism were expressed highly (Supplementary Table S2). The gene expressions related to malic acid pathway were shown in Table 2.

In Fig. 1B, the muC strain on CB1 and CB2 produced a lot more malic acid than that on CC1 and CC2. However, the transcriptions of the major genes related to malic acid synthesis were inconsistent with the fermentation results. We then further looked into the major genes related to the malic acid degradation to pyruvate, which were ‘malic’ enzyme (Tfu_0562) and malate dehydrogenase (oxaloacetate decarboxylating, Tfu_2390). Both genes on CC1 and CC2 transcribed significantly more highly than the one on CB1 and CB2, causing less malate accumulated by the muC strain on CC1 and CC2.

### PTS system related genes and the deletion of *hpr* (*Tfu_2487*)

The bacterial phosphoenolpyruvate (PEP): sugar phosphotransferase system (PTS) regulates the use of carbon sources in bacteria^14^,^15^,^16^. PTS system consists of enzyme I (EI) and histidine phosphocarrier protein (HPr) and several sugar specific enzyme IIs. EI transfers phosphoryl groups from PEP to the phosphoryl carrier protein HPr. HPr then transfers the phosphoryl groups to the different transport complexes. PTSs are ubiquitous in bacteria but do not occur in archaea and eukaryotes. It has been found that EI and HPr exist while enzyme IIs are absent in *T. fusca*^17^. And HPr (Tfu_2487) was thought to be the regulatory protein for *T. fusca*’s metabolism^17^.

On CB1 and CB2, there was no *Tfu_2487* transcription detected, while it transcribed highly on the medium on CC1 and CC2. The transcription of this gene was confirmed by RT-qPCR (Supplementary Table S1).

After searching the genome annotations of *T. fusca*, Tfu_2765 (enzyme I) and Tfu_2487 (HPr) have been well-annotated and they both transcribed highly (Supplementary Table S2). However, EII complexes, where the carbohydrate specificity of the PTS resides, were missing in *T. fusca*. The above results indicate that the PTS system was not complete in *T. fusca*, but it worked for the regulatory systems to control the carbon metabolisms^1^.

In order to determine if the zero transcription of *Tfu_2487* was the reason why the muC strain produced a lot more malic acid on yeast extract than ammonium sulfate, it was deleted in the muC strain by homologous recombination by using plasmid puC-hpr-del, forming strain muCΔ2487. The puC-hpr-del plasmid was not a replicating plasmid, which was eliminated after the homologous recombination.

**Figure 4:**
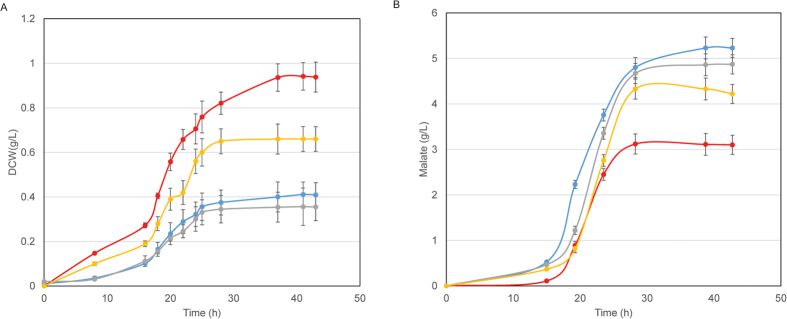
Fermentation results of *T. fusca* muC and muCΔ2487 strains. (**A**) Dry cell weight (DCW); (**B**) Malate production. The blue solid circles: muCΔ2487 on ammonium sulfate; the red solid circles: muC on ammonium sulfate; the gray solid circles: muCΔ2487 on yeast extract; the yellow solid circles: muC on yeast extract.

The muCΔ2487 and muC strains were then grown on the medium with ammonium sulfate or yeast extract, respectively. In Fig. 4, muCΔ2487 grown on ammonium sulfate had a little higher biomass (0.41 g/L) than yeast extract (0.35 g/L), however, it grew much worse than the muC strain (0.94 g/L DCW on ammonium sulfate and 0.41 g/L DCW on yeast extract). The highest titer of malate produced by muCΔ2487 was achieved on ammonium sulfate (5.23 g/L with 1.33 mole-malate/mole-glucose equivalent yield), not on yeast extract, and it was opposite to the muC strain (4.33 g/L on yeast extract with 1.10 mole-malate/mole-glucose equivalent yield). Besides, on either yeast extract or ammonium sulfate, muCΔ2487 produced a lot more malic acid than the muC strain (Fig. 4B)^18^. The above results confirm that HPr was the repressor for malic acid production

### Over expression of Tfu_2487 in muCΔ2487 strain

In order to study if the over-expression of *Tfu_2487* solely could affect cell growth and malic acid production, *Tfu_2487* was expressed in plasmid pYD-Tfu-hpr, a replicating plasmid designed for *T. fusca*.

**Figure 5:**
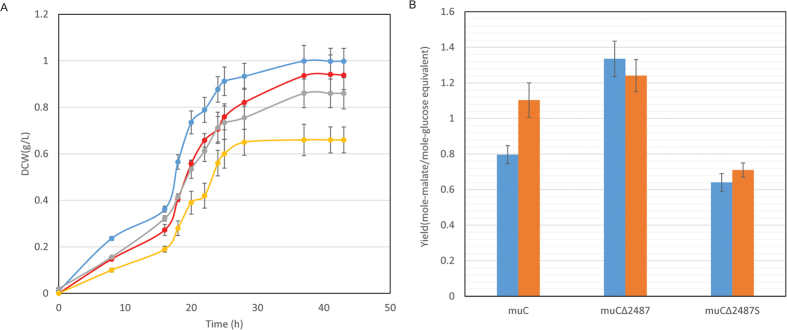
Fermentation results of *T. fusca* muC and muCΔ2487S strains. (**A**) Dry cell weight (DCW); (**B**) Malate yield. The blue solid circles: muCΔ2487 on ammonium sulfate; the red solid circles: muC on ammonium sulfate; the gray solid circles: muCΔ2487 on yeast extract; the yellow solid circles: muC on yeast extract; the blue bars: ammonium sulfate; the orange bars: yeast extract.

*Tfu_2487* gene was cloned into the *E. coli*-T.fusca shuttle plasmid pYD-Tfu-4 with thiostrepton resistant gene as the selection marker. The backbone of pYD-Tfu-4 was based on pIJ6021^19^, a replicating plasmid in *Streptomyces coelicolor* and puC18 in *E. coli*. However, the replication of origin of pYD-Tfu-4 was from *T. fusca* to ensure the replication in *T. fusca* strains. Besides, the promoter region for thiostrepton resistant gene (*tsr*) was replaced by the one for gapdh (*Tfu_2017*) transcription in *T. fusca* to ensure a strong transcription of *tsr* in *T. fusca*. The hpr gene was cloned to the multiple cloning site of this plasmid, forming pYD-Tfu-hpr. The transcription of *hpr* was controlled by a thiostrepton inducible promoter, tipA. The above plasmid replicated in muCΔ2487, forming the muCΔ2487S strain. The cell growth and malic acid yield of this mutant strain was shown in Fig. 5. The biomass of the muCΔ2487S strain was much higher than the muC strain both on ammonium sulfate (0.99 g/L for muCΔ2487S and 0.93 g/L for muC strain) and yeast extract (0.86 g/L for muCΔ2487S and 0.66 g/L for muC strain). The yield of malic acid by muCΔ2487S (0.64 mole-malate/mole-glucose equivalent) was much lower than the muC and muCΔ2487 strains (0.80 and 1.33 mole-malate/mole-glucose equivalent, respectively) on ammonium sulfate. Although yeast extract was thought to induce the production of malic acid, the malate yield of muCΔ2487S (0.71 mole-malate/mole-glucose equivalent) was significantly lower than muC (1.10 mole-malate/mole-glucose equivalent) and muCΔ2487 strains (1.24 mole-malate/mole-glucose equivalent). The above results indicate that the over-expression of Hpr could increase the biomass, however, the malate yield was reduced significantly.

## Discussion

*Thermobifida fusca* is a thermophilic actinobacterium. Previously, we isolated a mutant strain: *T. fusca* muC from adaptive evolution. *T. fusca* muC was confirmed to produce malic acid a lot more than its parent strain YX^10^. After identifying the malate synthesis pathway, we engineered the muC strain to produce a lot more malic acid from sugars^12^. Importantly, by fermentation optimization, the choices of nitrogen sources were found to affect the cell growth and malate production. However, the intracellular metabolisms related to the muC strain were largely unknown. In order to investigate the effect of nitrogen sources on cell growth and malate synthesis in *T. fusca* muC, RNAseq was employed to analyze the transcriptional changes corresponding to nitrogen sources. After analyzing the RNAseq data, the most important discovery was that *Tfu_2487*, annotated for histidine-containing protein (Hpr), didn’t transcribe on yeast extract, however it transcribed highly on ammonium sulfate. We then deleted *Tfu_2487* to get a mutant strain: muCΔ2487, which produced a lot more malic acid than the muC strain on ammonium sulfate (a nitrogen source bad for malate production by muC). However, the deletion of *Tfu_2487* is not the ultimate evidence to prove the role of Hpr in cell growth and malate production. We also need to over-express it in muCΔ2487, a strain without Hpr background. The *hpr* (*Tfu_2487*) gene was over-expressed on an *E. coli* -T. fusca shuttle plasmid pYD-Tfu-3 in muCΔ2487, forming muCΔ2487S. The muCΔ2487S strain experienced a low malate yield and faster cell growth compared to the muC strain.

HPr is an important member in the PTS system. We analyzed the genome annotations of *T. fusca*, and found that the PTS system was incomplete. In bacteria, HPr transfers a phosphoryl group to the CheY domains of response regulators, which typically regulate transcription. Adenylate cyclases increase the cellular level of cAMP, which, along with CAP protein, stimulates transcription from a number of promoters^14^,^15^,^16^,^20^. Adenylate cyclase, encoded by *Tfu_2552* in *T. fusca*, was found to be highly active. The proteins interacted with adenylate cyclase in *T. fusca* was predicted by STRING 10 (Supplementary Figure S3)^20^. Tfu_2552 might regulate RNA polymerase subunits (rpoB, rpoC, rpoA and rpoZ), which controlled the transcription of genes in *T. fusca*. The pyruvate kinase (Tfu_1179) was also regulated by Tfu_2552. Supplementary Figure S4 show that pyruvate kinase (Tfu_1179) interacted with malate dehydrogenase (Tfu_2390) and ‘malic enzyme’ (Tfu_0562), which are directly related to the synthesis of malic acid. Thus, the proposed rough scheme of malate regulation is: HPr → adenylate cyclase → pyruvate kinase → malate dehydrogenase and malic enzyme → malic acid. Thus, we will employ proteomics analysis and other experimental tools to verify and modify the above prediction.

Based on the fermentation data of the muCΔ2487 strain, the deletion of *hpr* increased the malic acid production significantly, indicating that HPr was the repressor for malic acid production. In addition, the over-expression of HPr in muCΔ2487, dramatically impaired the malic acid production, confirming the role of Hpr on malate synthesis. However, the mechanisms of HPr regulation in *T. fusca* need more study.

## Methods

### Strains and cultivation

*Thermobifida fusca* was grown on Hagerdahl medium with addition of desired carbon and nitrogen sources^2^,^21^. 10% V/V of pre-cultures of *T. fusca* were grown in the shaken flasks at 55 °C with 250 rpm rotation. The shaken flasks were sealed by the rubber stoppers to reduce the air exchange during the fermentation. 150 mL precultures were inoculated into a 5 L fermentor at 55 °C with various stirring speeds. *E. coli* was grown on Luria-Bertani (LB) or SOC medium for molecular work. The strains and plasmids used in this study are shown in Table 3. The primers and DNA sequences are shown in the Supplementary Table S2.

### Molecular Work

#### Deletion of Tfu_2487

The general strategy of deleting *Tfu_2487* in *T. fusca* was described previously^22^. The brief process is: the Hpr deletion cassette included ~500 bp DNA fragment homologous to the upstream of *hpr* gene, the inducible promoter region of *Tfu_2176* (endoglucanase) of *T. fusca* (genome location: 2,552,376-2,552,832, 457 bp), kanamycin resistant gene from pET-28a(+) (813 bp) and 500 bp DNA fragment homologous to the downstream of *hpr* gene (the sequences are shown in the supplementary file 1). The above cassette was synthesized and ligated to pUC57 by Genewiz (Suzhou, China), forming puC-hpr-del plasmid. The resulting plasmid with right structure was then transformed to *E. coli* BL21(DE3). Then, puC-hpr-del plasmid was introduced to *T. fusca* muC for deleting *hpr* gene.

#### Plasmid work

The backbone of the *E. coli*-T. fusca shuttle plasmid was puC18. The replication of origin of *T. fusca* was identified by the online tool (http://tubic.tju.edu.cn/doric/index.php)^23^,^24^. The DNA cassette: tfu-shuttle for *T. fusca* included: Kanamycin resistant gene, tsr-inducible MCS region, the gapdh promoter region of *T. fusca*, thiostrepton resistant gene (*tsr*) and origin of replication was synthesized by Genewiz (Suzhou, China). The puC18 was cut at NdeI and SacI and then was ligated to tfu-shuttle cassette by Gibson assembly^25^, which was then transformed to *E. coli* JM109. The transformants were picked from agar plates and subjected to colony PCR with primers hprEco-F and hprEco-R (Supplementary Table S1). The final plasmid was sequenced to verify the structure and designated as pYD-Tfu-4. The sequence and map of pYD-Tfu-4 are shown in Supplementary Figure S1.

#### Construction of the plasmid for expressing hpr in T. fusca

The *hpr* gene was amplified by primers hprF and hprR (Supplementary Table S1). The PCR product was cloned to pUCm-T vector (Sangon Biotech, Shanghai, China) and sequenced and digested by BamHI and SphI. The, the digested *hpr* gene was purified and ligated to pYD-Tfu-4, forming pYD-Tfu-hpr (Supplementary Figure S2).

### Transformation of *T. fusca* and allelic exchange

The puC-hpr-del and pYD-Tfu-4 plasmids were transformed to *T. fusca* protoplasts with the experimental details described previously^22^. The transformants with pYD-Tfu-4 were grown on kanamycin and thiostrepton. The deletion of hpr was confirmed by diagnosis PCR using primers hprdel-F and hprdel-R (Supplementary Table S1) and directly sequenced by Sanger sequencing, thus confirming a positive recombination event.

### Metabolite detection

The metabolites were detected by HPLC system (Dionex Ultimate3000) equipped with Bio-Rad HPX-87H ion exclusion column. The mobile phase was 0.005 mol/L H_2_SO_4_ at the rate of 0.6 mL/min using IR and UV detection.

### RNAseq work

*T. fusca* muC under different conditions was grown to 24 hours and then cells were harvested by centrifugation at >10,000 g for 15 min. The cell pellets were washed by fresh medium and then spin down again to get rid of the supernatant. The above process was repeated three times. The general procedures about sample preparation for RNAseq analysis were described previously^26^. An Illumina HiSeq2000 PE100 was used in this study.

The biological duplicates were used in this study. SRA accession number for samples is SRP067532.

### Statistical Analysis

The Unity platform developed by Genome Canada was used to process the raw data. In this study, the reported data were subject to log2-transformation^26^. If log_2_(a) − log_2_(b) (defined as log2 fold change or LFC) ≥ 1 or ≤−1, the difference between a and b was significant. The other statistical analysis methods were according to Deng *et al*.^26^.

### RT-PCR

The RT-qPCR experiments were describe previously^21^,^22^,^27^ and the primers used are shown in Supplementary Table S1.

### Cellulase activity

The general process of measuring cellulase activity has been described previously^22^.

## Additional Information

**How to cite this article**: Deng, Y. *et al*. Systematic analysis of an evolved *Thermobifida fusca* muC producing malic acid on organic and inorganic nitrogen sources. *Sci. Rep.* **6**, 30025; doi: 10.1038/srep30025 (2016).

## Supplementary Material

Supplementary Information

Supplementary Table S2

## Figures and Tables

**Table 1:** The cellulase and hemicellulase gene expression changes.

Cellulase	Definitions	CC1-CB1	CC2-CB2
Tfu_0620	cellobiohydrolase (EC:3.2.1.91)	−1.08	1.59
Tfu_0901	endoglucanase [EC:3.2.1.4]	0.75	1.13
Tfu_0937	beta-glucosidase [EC:3.2.1.21]	−1.73	2.79
Tfu_1074	endoglucanase [EC:3.2.1.4]	0.12	1.78
Tfu_1607	exo-1,4-beta-glucosidase (EC:3.2.1.74)	0.74	−2.17
Tfu_1627	cellulose 1,4-beta-cellobiosidase [EC:3.2.1.91]	0.49	1.61
Tfu_1959	cellulose 1,4-beta-cellobiosidase [EC:3.2.1.91]	−0.05	1.82
Tfu_2176	endoglucanase [EC:3.2.1.4]	1.09	2.12
Tfu_2712	endoglucanase [EC:3.2.1.4]	0.08	2.38
Tfu_1629	beta-glucosidase (EC:3.2.1.21)	1.36	1.26
Hemicellulase			
Tfu_1213	endo-1,4-beta-xylanase [EC:3.2.1.8]	0.79	3.23
Tfu_2791	endo-1,4-beta-xylanase [EC:3.2.1.8]	1.39	3.83
Tfu_2923	endo-1,4-beta-xylanase [EC:3.2.1.8]	−0.14	2.59

Note: LFC: log fold change. When LFC ≥ 1 or LFC ≤ −1, the changes are significantly different. CB1: 2 g/L yeast extract, 5 g/L cellobiose; CB2: 2 g/L yeast extract, 5 g/L Avicel; CC1: 2 g/L ammonium sulfate, 5 g/L cellobiose; CC2: 2 g/L ammonium sulfate, 5 g/L Avicel.

**Table 2:** The expression changes of malic acid related genes.

Gene	Definition	CC1-CB1	CC2-CB2
Tfu_0092	malate dehydrogenase	−1.32	2.53
Tfu_0562	‘malic’ enzyme	1.63	3.49
Tfu_2390	Malate dehydrogenase (Oxaloacetate decarboxylating)	2.04	2.42
Tfu_0459	Fumarate hydratase	−0.58	1.35
Tfu_2451	Succinate dehydrogenase	−0.51	1.54
Tfu_2452	Succinate dehydrogenase	−0.82	1.37
Tfu_0083	Phosphoenolpyruvate carboxykinase	2.33	1.54

Note: LFC: log fold change. When LFC ≥ 1 or LFC ≤ −1, the changes are significantly different. CB1: 2 g/L yeast extract, 5 g/L cellobiose; CB2: 2 g/L yeast extract, 5 g/L Avicel; CC1: 2 g/L ammonium sulfate, 5 g/L cellobiose; CC2: 2 g/L ammonium sulfate, 5 g/L Avicel.

**Table 3:** Strains and plasmids used in this study.

Strain	Genotype/comments	Source/Ref.
*Thermobifida fusca* muC	Mutant stain obtained by adaptive evolution	10
*Thermobifida fusca* muCΔ2487	*ΔTfu_2487* in the muC strain	This study
*Thermobifida fusca* muCΔ2487S	Over-expression of *Tfu_2487* in the muCΔ2487 strain	This study
*E. coli* JM109	*endA1 glnV44 thi-1 relA1 gyrA96 recA1 mcrB*+ *Δ*(*lac-proAB*) *e14- [F*’ *traD36 proAB*+ *lacIq lacZΔM15] hsdR17*(*rK-mK*+)	Gift from Professor Jingwen Zhou of Jiangnan University
*E. coli* BL21	*fhuA2 [lon] ompT gal sulA11 R*(*mcr-73::miniTn10*–Tet^S^*)2 [dcm] R(zgb-210::Tn10*–Tet^S^*) endA1 Δ(mcrC-mrr)114::IS10*	Gift from Professor Jingwen Zhou of Jiangnan University
Plasmid	Genotype/comments	Source/Ref.
pUCm-T	T-vector	Sangon Biotech
puC57m	a common used plasmid cloning vector in *E. coli*	Sangon Biotech
puC-hpr-del	Plasmid used for deleting *hpr* in T. fusca	This study
pYD-Tfu-4	T. fusca-*E. coli* shuttle plasmid	This study
pYD-Tfu-hpr	pYD-Tfu-3 plasmid carrying *hpr* gene	This study
