# Supplementary material for: Systematic analysis of an evolved Thermobifida fusca muC producing malic acid on organic and inorganic nitrogen sources
Source: Sci Rep. 2016 Jul 18;6:30025. doi: 10.1038/srep30025 (PMC4948018; doi:10.1038/srep30025)
Supplement: Supplementary Information [file srep30025-s1.doc]

**Systematic analysis of an evolved *Thermobifida fusca* muC producing malic acid on organic and inorganic nitrogen sources**

Yu Deng^1,2*^, Jia Lin^4^, Yin Mao^1,2^ and Xiaojuan Zhang^3*^

1 National Engineering Laboratory for Cereal Fermentation Technology (NELCF), Jiangnan University, 1800 Lihu Road, Wuxi, Jiangsu 214122, China.

2 The Key Laboratory of Industrial Biotechnology, Ministry of Education, Jiangnan University, 1800 Lihu Road, Wuxi, Jiangsu 214122, China.

3 School of pharmaceutical science, Jiangnan University, 1800 Lihu Road, Wuxi, Jiangsu 214122, China.

4 College of Life Science, North China University of Science and Technology, Tangshan 063000, China

Running title: Regulation of *T. fusca* on nitrogen sources

*Corresponding authors:

Yu Deng, Ph.D, National Engineering Laboratory for Cereal Fermentation Technology (NELCF), Jiangnan University, 1800 Lihu Road, Wuxi, Jiangsu 214122, China. Tel: +86-510-85916977; Fax: +86-510-85918309; E-mail: dengyu@jiangnan.edu.cn

Xiaojuan Zhang, Ph.D, School of pharmaceutical science, Jiangnan University, 1800 Lihu Road, Wuxi, Jiangsu 214122, China. E-mail: xzhang6@163.com

Table S1 Primers used in this study

| ID | Sequence | Comments |
| --- | --- | --- |
| hprF | gcatgcGTGAGCCGCGCGGAACGCCGGGT (SphI) | Forward primer for amplifying hpr gene |
| hprR | ggatccTCACGGCGTAGCGGCGATCT (BamHI) | Reverse primer for amplifying hpr gene |
| hprEco-F | CTATTTCTGAATGACGTAAATCAGC | Forward primer for picking up transformants with pYD-Tfu-4 |
| hprEco-R | ATGGTTTCTTAGTCGCGTTC | Reverse primer for picking up transformants with pYD-Tfu-4 |
| hprdel-F | ATCGTGGTGTTCGAGGACA | Forward primer for detecting the deletion of hpr gene |
| hprdel-R | TTTCACAGACTAAGGATGACGAC | Reverse primer for detecting the deletion of hpr gene |
| hprRT-1F | GCGGTTCGTTACGGCGGCCT | Forward primer for RT-qPCR |
| hprRT-1R | CACGGCGTAGCGGCGATCTC | Reverse primer for RT-qPCR |

**Hpr deletion cassette**

**Upstream**

>gi|72160406:c2929936-2929437 *Thermobifida fusca* YX, complete genome

CGGGTCTGTCCCGGCGCTGCCGACCGCGACCGCGGCCCGGAGAACGACGCGTCGTCATCCTTAGTCTGTGAAAGCGTGCCCATGTTTCGACGTCTGCCTGTGCTGGCCGGTGCAACCGTTCTGCTGTTCACAACCGCCTGCGGCGGGGGGAGCGCACCCCGCCCGGGGGAGCGCACCACCCAGATATCCGACCCCGCTGACGTGGACGCTCAGGCGGCAGCGCTGCTCGCGGAGATGAGCCTGGAAGAGAAAGTGGGACAGCTGCTGGTCCCCGTACTCTCCGGCACCACGGCCGAGGAGAACGCCGAGGTGATCGAGCGCTACCACCCAGGCGGGTTCATCTACTTCCCCGAGAACTTGGAGACTCCCGAGCAGGTGGCTGCCATGTCCAACGGGCTGCAAGAGCGTGCCACCGGCACGGGGGCGGGGATTCCGCTGTTCCTCGGGGTGGACGAGGAGCAGGGACTGGTGTCGCGGCTCCCGTTCGGTGCCCGTTTCCC

**The inducible promoter region of *Tfu_2176* (endoglucanase) of *T. fusca* (genome location: 2,552,376-2,552,832, 457 bp)**

ggtgtcttcg ctttctgcag tcggttgtcc tacacaccgc tttggacggc gcgggtagcggcggtccgct ccccggatcg gagcccagcg ccgcagacgg tggggtccgg atgtgttccgccgtcccacg cacaacggta tcggcgtcgc ggggccgccg ccactgcagc tgtggggaggcggtctgtgg gccgccgggc acggcgcccg ggccacgtgc ggggccgcac cctgggcgggcagggatgcc gcggcggccg ctccgacggg cagcggcagc agatctcccc cgctgctccg

tgtcagccgc acgcgaaggg cgactactta aggttcaccc attgacgcaa tcggtcaccacgtcgcacca tgctggagac gtcacttcac agagagccaa caccaaactg ggagcgctcccacaccggtg tgttcgcaca atccccctgg agacccc

**Kanamycin resistant gene from pET-28a(+) (813 bp)**

ttagaaaaactcatcgagcatcaaatgaaactgcaatttattcatatcaggattatcaataccatatttttgaaaaagccgtttctgtaatgaaggagaaaactcaccgaggcagttccataggatggcaagatcctggtatcggtctgcgattccgactcgtccaacatcaatacaacctattaatttcccctcgtcaaaaataaggttatcaagtgagaaatcaccatgagtgacgactgaatccggtgagaatggcaaaagtttatgcatttctttccagacttgttcaacaggccagccattacgctcgtcatcaaaatcactcgcatcaaccaaaccgttattcattcgtgattgcgcctgagcgagacgaaatacgcggtcgctgttaaaaggacaattacaaacaggaatcgaatgcaaccggcgcaggaacactgccagcgcatcaacaatattttcacctgaatcaggatattcttctaatacctggaatgctgttttcccggggatcgcagtggtgagtaaccatgcatcatcaggagtacggataaaatgcttgatggtcggaagaggcataaattccgtcagccagtttagtctgaccatctcatctgtaacatcattggcaacgctacctttgccatgtttcagaaacaactctggcgcatcgggcttcccatacaatcgatagattgtcgcacctgattgcccgacattatcgcgagcccatttatacccatataaatcagcatccatgttggaatttaatcgcggcctagagcaagacgtttcccgttgaatatggctcat

**Downstream**

>gi|72160406:c2930438-2929938 Thermobifida fusca YX, complete genome

TCAGCGGAGCCGAGCTTGTCGGCTACGGCCCCGAGCACATCGTGGTGTTCGAGGACACCCCGGCGGGGATCATGGCGGGGCGCAACGCGGGGATGCGGGTCGTCGGAGTCACGACGACGCATCCGCCGCAGGCGCTGGCCCACGCCCATCTCGTCGTGGAGCATCTGGGACAGGTGGGCTGGCCGCAGTTGGTTTTGCGGGATCCGGAGCCGCCCCAGACTGCTCTGGCAGGTCAAGGGTGAGCCGCGCGGAACGCCGGGTCACCGTGGCTTCCCCCGTGGGACTGCATGCCCGGCCCGCGGCGCGGTTCGTTACGGCGGCCTCCGAGTTCGACGGGGAGGTGCGCATTGCCCGTCCCAGCGGGGCGAGCGTGCCGGCGACGAGCATGCTGGCGGTGCTGGCGCTGGGGGTGCGCTGCGGGGAGGAGATCGTGATCACCGCGGAGGGCGAGGGGGCCGAAGAGCTGCTGGACCGTCTCGCCGAGATCGCCGCTACGCCGTG

*Tfu_2487 (hpr)*

>gi|72160406:c2930200-2929937 Thermobifida fusca YX, complete genome

GTGAGCCGCGCGGAACGCCGGGTCACCGTGGCTTCCCCCGTGGGACTGCATGCCCGGCCCGCGGCGCGGTTCGTTACGGCGGCCTCCGAGTTCGACGGGGAGGTGCGCATTGCCCGTCCCAGCGGGGCGAGCGTGCCGGCGACGAGCATGCTGGCGGTGCTGGCGCTGGGGGTGCGCTGCGGGGAGGAGATCGTGATCACCGCGGAGGGCGAGGGGGCCGAAGAGCTGCTGGACCGTCTCGCCGAGATCGCCGCTACGCCGTGA


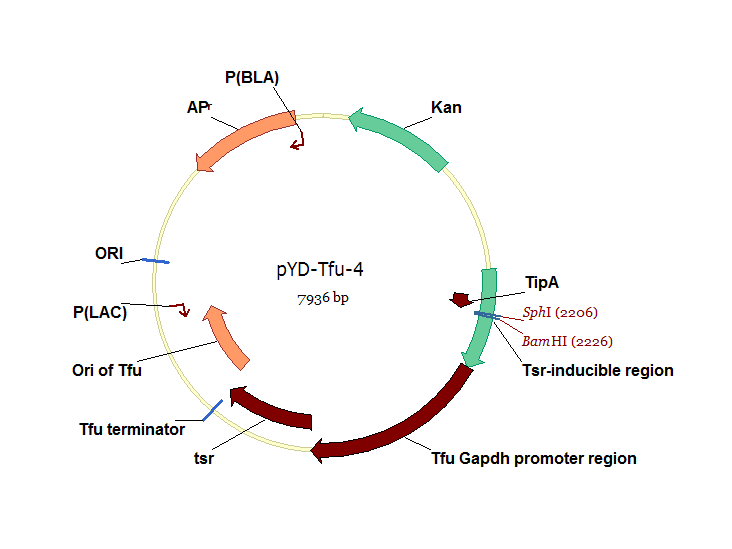


Figure S1 Map of the E.coli -T. fusca shuttle plasmid pYD-Tfu-4

**Sequence:**

tcgcgcgtttcggtgatgacggtgaaaacctctgacacatgcagctcccggagacggtcacagcttgtctgtaagcggatgccgggagcagacaagcccgtcagggcgcgtcagcgggtgttggcgggtgtcggggctggcttaactatgcggcatcagagcagattgtactgagagtgcaccatatgctatttctgaatgacgtaaatcagctcgttgccgatctccagtcgctgaatgcggcacgaccgctctctggcctgggactcaaaactttgtgaatagttctgaaacatccccttcgatcgctgaccgagagacttggtcgggaaggttaccacgataatcggcgagttgacaatgtcaatcacttcccagccggagcctcgtcgctgagtctccagacaggggagcgtcttcagcaatagcgtgacgtcggtcggttcgtcaaggcggtcctcaaggacgtcgaccacactcgtgcggtgcgcgacgcccaacctcgtcagggcggcgtccacgaagtcgatcagccgggcgtcgatgtcggaggcgacgtagacggtctcgtccgacaggcccatccacggggcggccagcgggttgaggccgcaggcaaggtcacgcagcgtgttgggccgggggacgtggcggaagacctcccggtagaactccgccaggtggggcaatcgttcgcgggtggacacgtgcaccgacatcgcgcgccgcagggccgcctggaccgccgcgtcgtcaccgtcgtccacagcggagtcgagctgccggagcaacgctgcgtagttgggcggactgggcggcaggaaggcgccgtagatctcgtgcagcccgcgcttggtgcgcttcaccgcgtccggcacgtcgccccgcgtggcgacgagggcggcgcgggccagccgccgcacggtggccggggccaccgtctggtagcgccggctcttggtgatggcctgctcgacctgctcgatacggtcgtcgggcacagatgtcgtcatcgaatcctccgaaagatcatgaaccggcagcgaagcggaaaaattcccccaccaatacccgctccgcacaccaagagtcattgccacgtgtacttaaagttattgcaggagtattaccgggcgggcatgggagcgctcgcagcccgcccctcgtcacgtccatattggtcggcctgtcctgcaagcccggcttggccggtttttgcacgtcgacactggtgattgtgtctggcacacttggccggggccgcgatcctcgccgagcaggccgtcgaggacatcttccccctcccccgcgagctggtgggcgagggcgaggtcttcatgctccaggtcaagggcgactcgatgctcgacgccgccatctgcgacggggactgggtcgtggtccggcagcagcccaccgccgacagcggcgagatctacaagggccgccggatacagggccggacgtcaccgacggcgggggccaccacggcggcggtcacggcggtgacggtcacggcggcggccatcacggcggcggttacgccgtgttcgtggacggcgtcgaactgcatgtgatgcgcaacgccgacggctcgtggatcagcgtcgtcagccactacgagccggtggacaccccgcgcgccgcggcccgcgctgcggtcgacgagctcggtacccggggatctgtgttggcgcacaaatcaacggggattactgtcgtttaatgtgatttaaactgtgaaatagtatggttttcagttattgaaacgccgtgagcggggaaaacttgctttttcccgtttccggggttggacaactgagcaacgcgaaggcgtcagctacgatgttccggggactgctgatccggtcagcaggtggaagagggactggattccaaagttctcaatgctgcttgctgttcttgaatggggggtcgttgacgacgacatggctcgattggcgcgacaagttgctgcgattctcaccaataaaaaacgcccggcggcaaccgagcgttctgaacaaatccagatggagttctgaggtcattactggaccggatcggggatctgggctgagggagccgacggcacgcggcggctcacggcgtggcacgcggaacgtccgggcttgcacctcacgtcacgtgaggaggcagcgtggacggcgtcagagaagggagcggacatatgaagcttgcatgcctgcaggtcgactctagaggatccccgggtaccgagctcgaattccccagatctaaagttttgtcgtctttccagacgttagtaaatgaattttctgtatgaggttttgctaaacaactttcaacagtttcagcggagtgagaatagaaaggaacaactaaaggaattgcgaataataattttttcacgttgaaaatctccaaaaaaaaaggctccaaaaggagcctttaattgtatcggtttatcagcttgctttcgaggtgaatttcttaaacagcttgataccgatagttgcgccgacaatgacaacaaccatcgcccacgcataaccgatatattcggtcgctgaggcttgcagggagtcaaaggccgcttttgcgggatctcgtcgaaggcggcgggggcgccggacgcggccgggttcccgggtgtgaggatgatcacaatatatatccgagagtagtcatttggtcactctcggatagggcgtggggtggtagaaggggaaagtagcccaatggccggtcaacatccctcgttaatgcgcccgtaataaagtgtgaacctttttctggaaatgcccacactttcacgagctgcggaaacaatggtggtgggtggggaagaatacggtctaatggtctatacctttgaggtgtgagggtgccagtattggccccgacgggacagcccgcgccgtagcagatccggctccggcggctcggaacgcggacgataggctcgccccgacgaaacgcacactgttttccacggtccgacgcgtacgcgtcggaaacaagcgtcgacgacgcagacgacacgtgaggagaatggttccgtgaccatccgtgtaggtgtaaacggattcggacgaatcggccgtaacttctggcgggcggttcaggccgccggcggcagcgatgtcgaaatcgtggcggtcaacgacctcaccgacaaggccaccctggcgcacctgctcaagtacgacacggtgctgggcaccctgccgggcgaggtcgaggtcggcgaggacagcatcacggtcggcggcaccaccatgaaggcgctcgcccagcgcgacccggctcagctcccgtggggcgacctcggggtggacatcgtcgtggagtccactggcttcttcaccaaggctgaggacgcgaagaagcacctcgacgccggcgccaagaaggtcatcatctccgcccccgccaagggcgaggacctgacggtcgtcatgggcgtcaacgacgacaagtacgacccggccaaccaccacatcctgtccaacgcctcctgcaccaccaactgcgtggcgccgatggcgaagaccctgatggagaacttcggcatcgtcaagggtctgatgaccacggtgcacgcctacaccaacgaccaggtcatcctggactacccgcacaaggacctgcgccgtgcccgggccgccgcgcagaacatcatcccgaccaccaccggtgccgccaaggccaccgctctggtgctgcccgagctgaagggcaagctcgacggcctggcgatgcgggtgccggtgccggacggctcggtgaccgacctggtggtcacgctggagcgcgaagtcaccaaggaagaggtcaacgccgccttcaaggctgcggccgagggcgcgctcaaggacatcctggtctacaccgaggacccgatcgtctcctccgacatcgtcggcaccccggcgtcctgcaccttcgacgccagcctcaccatggccttcggcacccaggtcaaggtcgtcggctggtacgacaacgagtggggctactccaaccgcctggtcgacctggtcaagctggtcggctccaacctctgatgactgagttggacaccatcgcaaatccgtccgatcccgcggtgcagcggatcatcgatgtcaccaagccgtcgcgatccaacataaagacaacgttgatcgaggacgtcgagcccctcatgcacagcatcgcggccggggtggagttcatcgaggtctacggcagcgacagcagtccttttccatctgagttgctggatctgtgcgggcggcagaacataccggtccgcctcatcgactcctcgatcgtcaaccagttgttcaagggggagcggaaggccaagacattcggcatcgcccgcgtccctcgcccggccaggttcggcgatatcgcgagccggcgtggggacgtcgtcgttctcgacggggtgaagatcgtcgggaacatcggcgcgatagtacgcacgtcgctcgcgctcggagcgtcggggatcatcctggtcgacagtgacatcaccagcatcgcggaccggcgtctccaaagggccagccgaggttacgtcttctcccttcccgtcgttctctccggtcgcgaggaggccatcgccttcattcgggacagcggtatgcagctgatgacgctcaaggcggatggcgacatttccgtgaaggaactcggggacaatccggatcggctggccttgctgttcggcagcgaaaagggtgggccttccgacctgttcgaggaggcgtcttccgcctcggtttccatccccatgatgagccagaccgagtctctcaacgtttccgtttccctcggaatcgcgctgcacgagaggatcgacaggaatctcgcggccaaccgataacacgccctatccgagagtgaccaaatgactactctcggatatatattgtgaacgggcgtcgggggtggaccgccacccccgacgcccgttcacgggctggtcagggggaggtcactgcactccccaggggtgtgaaaccactcacttcgcggcggttcgccccggactttccccctgttttccccatgcccctcccgcggccctataccacatcccaagctgtggataagccgtggataactctggggataactcccaattttctgtggacagcctggggatacgctgtgtataacccgccacgacatccagtcatcccatgaggggatctgtgggtaaagtcgtggacaagctgtggacaacctggggatgacttggggacaaccgcgctctgtccacaggacgccgagttttccacacccgccatccacatctcctgtggacaaaaatcaccgagctgacctgcgaaaacagggtttgtccacagtatccacagcccctattactactgcgccacaaagagagatcttcgtggcttagtaaacgggctcggccgatttctgtggacaacgctccccgctgccccaacttctgtccacaactctccttctcggagagtcccctccgactcgacgcctcgaccgtcgtacactcgcgcccgaataggggtaccgtggtgaactcacccccgatgtcctgaccgacatcccgacgcggacggtcccgtccgttgacccgcaagcgccgacacgtgcgacccagggcgtcggtcggcggttcatagtcgtcgaggaaagagagaccgatcgtgaagttccgggtcgaacgcgacgagctcgaattcgtaatcatggtcatagctgtttcctgtgtgaaattgttatccgctcacaattccacacaacatacgagccggaagcataaagtgtaaagcctggggtgcctaatgagtgagctaactcacattaattgcgttgcgctcactgcccgctttccagtcgggaaacctgtcgtgccagctgcattaatgaatcggccaacgcgcggggagaggcggtttgcgtattgggcgctcttccgcttcctcgctcactgactcgctgcgctcggtcgttcggctgcggcgagcggtatcagctcactcaaaggcggtaatacggttatccacagaatcaggggataacgcaggaaagaacatgtgagcaaaaggccagcaaaaggccaggaaccgtaaaaaggccgcgttgctggcgtttttccataggctccgcccccctgacgagcatcacaaaaatcgacgctcaagtcagaggtggcgaaacccgacaggactataaagataccaggcgtttccccctggaagctccctcgtgcgctctcctgttccgaccctgccgcttaccggatacctgtccgcctttctcccttcgggaagcgtggcgctttctcatagctcacgctgtaggtatctcagttcggtgtaggtcgttcgctccaagctgggctgtgtgcacgaaccccccgttcagcccgaccgctgcgccttatccggtaactatcgtcttgagtccaacccggtaagacacgacttatcgccactggcagcagccactggtaacaggattagcagagcgaggtatgtaggcggtgctacagagttcttgaagtggtggcctaactacggctacactagaaggacagtatttggtatctgcgctctgctgaagccagttaccttcggaaaaagagttggtagctcttgatccggcaaacaaaccaccgctggtagcggtggtttttttgtttgcaagcagcagattacgcgcagaaaaaaaggatctcaagaagatcctttgatcttttctacggggtctgacgctcagtggaacgaaaactcacgttaagggattttggtcatgagattatcaaaaaggatcttcacctagatccttttaaattaaaaatgaagttttaaatcaatctaaagtatatatgagtaaacttggtctgacagttaccaatgcttaatcagtgaggcacctatctcagcgatctgtctatttcgttcatccatagttgcctgactccccgtcgtgtagataactacgatacgggagggcttaccatctggccccagtgctgcaatgataccgcgagacccacgctcaccggctccagatttatcagcaataaaccagccagccggaagggccgagcgcagaagtggtcctgcaactttatccgcctccatccagtctattaattgttgccgggaagctagagtaagtagttcgccagttaatagtttgcgcaacgttgttgccattgctacaggcatcgtggtgtcacgctcgtcgtttggtatggcttcattcagctccggttcccaacgatcaaggcgagttacatgatcccccatgttgtgcaaaaaagcggttagctccttcggtcctccgatcgttgtcagaagtaagttggccgcagtgttatcactcatggttatggcagcactgcataattctcttactgtcatgccatccgtaagatgcttttctgtgactggtgagtactcaaccaagtcattctgagaatagtgtatgcggcgaccgagttgctcttgcccggcgtcaatacgggataataccgcgccacatagcagaactttaaaagtgctcatcattggaaaacgttcttcggggcgaaaactctcaaggatcttaccgctgttgagatccagttcgatgtaacccactcgtgcacccaactgatcttcagcatcttttactttcaccagcgtttctgggtgagcaaaaacaggaaggcaaaatgccgcaaaaaagggaataagggcgacacggaaatgttgaatactcatactcttcctttttcaatattattgaagcatttatcagggttattgtctcatgagcggatacatatttgaatgtatttagaaaaataaacaaataggggttccgcgcacatttccccgaaaagtgccacctgacgtctaagaaaccattattatcatgacattaacctataaaaataggcgtatcacgaggccctttcgtc


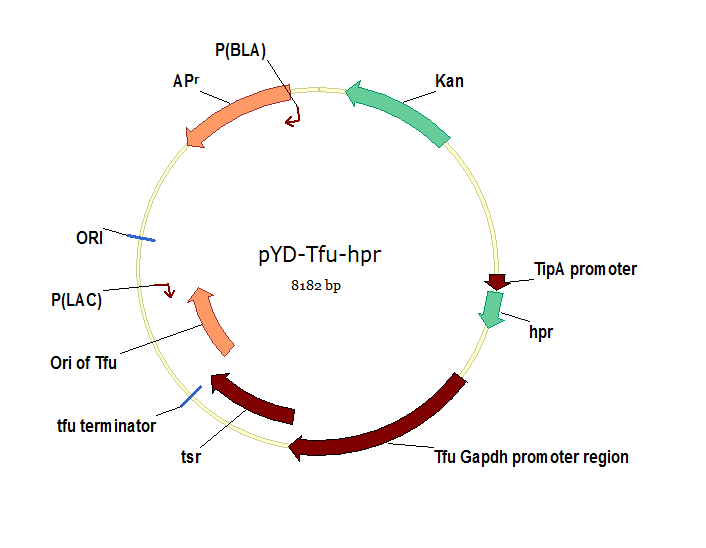


Figure S2 Map of the E.coli-T. fusca shuttle plasmid pYD-Tfu-hpr harboring *hpr* gene


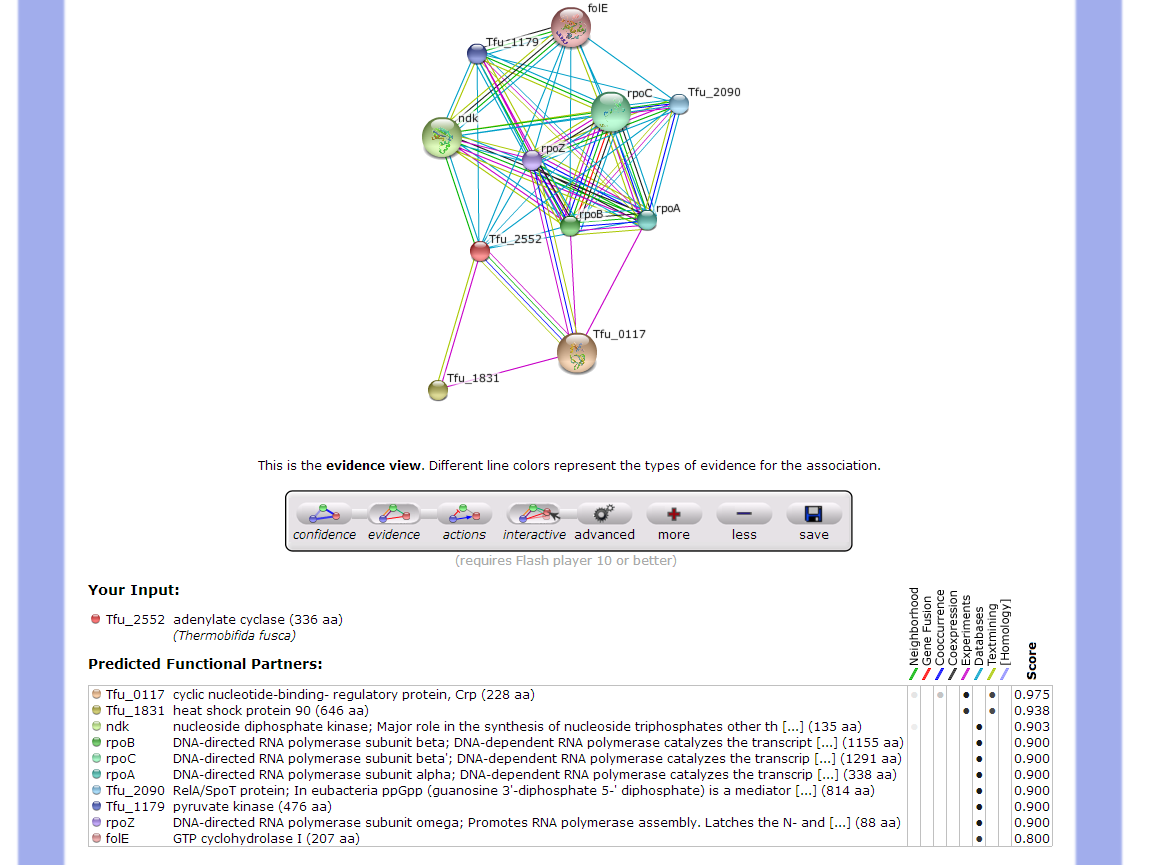


Figure S3 The proteins predicted by STRING 10[^1^](#_ENREF_1) to be interacting with Tfu_2552


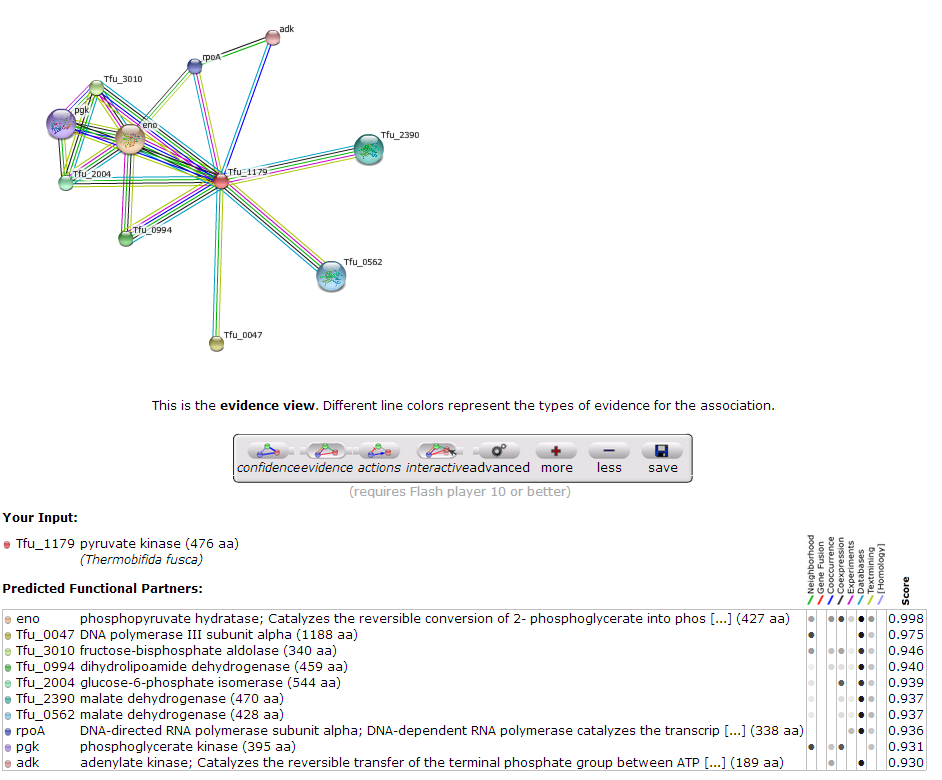


Figure S4 The proteins predicted by STRING 10[^1^](#_ENREF_1) to be interacting with Tfu_1179

1 Szklarczyk, *D. et a*l. STRING v10: protein-protein interaction networks, integrated over the tree of life*. Nucleic Acids R*e**s** 43, D447-452, doi:10.1093/nar/gku1003 (2015).
